# The interplay of daily affect and impulsivity measured by mobile surveys in bipolar disorder

**DOI:** 10.1186/s40345-022-00270-8

**Published:** 2022-10-31

**Authors:** Madison K. Titone, Colin Depp, Federica Klaus, Jessica Carrasco, Jared W. Young, Lisa T. Eyler

**Affiliations:** 1 UC San Diego Department of Psychiatry, 9500 Gilman Drive, La Jolla, CA 92093 USA; 2 VA San Diego Healthcare System, 3350 La Jolla Village Drive, San Diego, CA 92161 USA; 3 Desert-Pacific Mental Illness Research Education and Clinical Center, Department of Psychology, VA San Diego Healthcare System, 3350 La Jolla Village Drive, San Diego, CA 92161 USA; 4 Department of Psychology, San Diego State University, 5500 Camponile Drive, San Diego, CA 92182 USA

**Keywords:** Bipolar disorder, Affect, Impulsivity, Ecological momentary assessment, Time-lagged analysis

## Abstract

**Background:**

Impulsivity is a prominent feature of bipolar disorder associated with various negative sequelae; moreover, it may be a precursor to shifts in affect or mood, but little is known about its association with affect on a day-to-day timescale. Ecological momentary assessments (a method that captures moment-to-moment ratings of psychological states by repeatedly sampling the same individual) of impulsivity and affect using mobile surveys allow for more nuanced examination of mechanisms of mood and behavior dysregulation. However, few existing studies have validated an ecological momentary assessment of impulsivity in bipolar disorder and examined its time-lagged associations with positive and negative affect. 70 participants with bipolar disorder and 102 healthy comparisons participated in an intensive longitudinal study: they underwent 14 days of ecological momentary assessment data collection annually for 1–4 years. Multiple measures of impulsivity and affect were collected using self-report, behavioral, and ecological momentary assessment modalities; these measures were compared, and levels of impulsivity were compared between bipolar disorder and healthy comparison groups. Time-lagged analyses using daily means explored the next-day predictive relationship of impulsivity on positive/negative affect, and vice versa.

**Results:**

The ecological momentary measure of impulsivity was moderately correlated with the self-report but not behavioral impulsivity measure. Bipolar disorder participants evinced higher self-report, behavioral, and daily impulsivity than healthy comparison participants. Time-lagged analyses revealed a bi-directional association between high impulsivity and high next-day negative (but not positive) affect. Post hoc analyses showed that impulsivity specifically predicted next-day anger and anxiety.

**Conclusions:**

Our multimodal assessment of impulsivity allowed for an examination of the day-to-day course of impulsivity and affect, crucial steps toward understanding the mechanisms of mood symptom and episode onset in bipolar disorder.

## Background

Impulsivity is a prominent feature of bipolar disorder (BD) that persists even between mood episodes (Strakowski et al. 2010; Swann et al. 2009) and predicts lower quality of life, higher functional impairment, longer duration of illness, and a greater number of suicide attempts (Ekinci et al. 2011; Jiménez et al. 2012; Victor et al. 2011). Given impulsivity’s core role in BD and its association with the more severe consequences of the illness, it is crucial to understand its temporal relationship to mood states and changes in affect, and to effectively measure these elements in the context of BD.

Whereas *impulsive behavior* is a core symptom of mania in the Diagnostic and Statistical Manual of Mental Disorders, 5th edition (DSM-5), *impulsivity,* or the predisposition toward action without forethought, is elevated across mood states in BD (Strakowski et al. 2010; Swann et al. 2009; American Psychiatric Association 2013; Newman and Meyer 2014; Saddichha and Schuetz 2014). Impulsivity is a complex and multidimensional construct with no widely agreed-upon factor structure (Sharma et al. 2013). Researchers have attempted to categorize impulsivity using many different paradigms; past studies have made distinctions between impulsive traits and states (Halvorson et al. 2021), between behavioral and self-reported impulsivity (Sharma et al. 2013), and between impulsive choice and impulsive action (McCarthy et al. 2018). Various measures for capturing self-reported impulsivity have been investigated, including the Barratt Impulsiveness Scale (Barratt 1959; Patton et al. 1995). Impulsive states also have been measured via various behavioral tasks, broadly separated into tasks measuring impulsive choice (delay discounting, or the ability to delay present gratification for larger future rewards) and impulsive action (behavioral inhibition, or the inability to inhibit a pre-potent response) (Halvorson et al. 2021). One method for measuring behavioral inhibition is examining the number of false alarms (non-target responses) on a Continuous Performance Task (CPT) of sustained/selective attention; one adaptation of a CPT found severe task deficits for manic BD patients and moderate task deficits for euthymic BD patients (Young et al. 2020).

Additional complexity stems from the findings that laboratory measures of self-report and behavioral impulsivity do not correlate well with one another and correlate only moderately with real-life impulsive behaviors (e.g., aggression, delinquency, gambling, substance use) (Sharma et al. 2013; Cyders and Coskunpinar 2011). Thus, new types of ecologically valid measures may be needed to capture momentary or state impulsivity; ecological momentary assessment (EMA) is a method of capturing moment-to-moment “snapshots” of psychological states by repeatedly sampling the same individual. This method may provide more meaningful and externally valid data compared to traditional self-report and behavioral methods (Shiffman et al. 2008).

In spite of the enormous potential of EMA methods, few studies have established and validated EMA paradigms for measuring impulsivity. Multiple studies (the review of which are beyond the scope of this paper) have measured momentary impulsivity by adapting existing retrospective personality, self-report, or behavioral measures (aan het Rot et al. 2015; MacLean et al. 2018; Sperry et al. 2018), but few of these measures have been validated against other impulsivity measures or shown discriminant or predictive validity in relation to mental health outcomes. Amongst those EMA impulsivity measures that have been validated (Sharma et al. 2013; Halvorson et al. 2021; Wu and Clark 2003; Tomko et al. 2014), results generally showed that the EMA-adapted measures of impulsivity had a similar structure to the traditional self-report measures and had high criterion validity in clinical samples. Thus, evidence from the few existing validated studies suggests that EMA-measured impulsivity has a moderate correlation to global self-report measures.

Nevertheless, it is unclear how EMA-measured impulsivity correlates to more traditional self-report and behavioral measures of impulsivity in the context of BD. Understanding the nature of moment-to-moment impulsivity and creating ecologically valid measures may be important particularly in BD; impulsivity and affect are both dynamic processes that change rapidly and may influence one another over the course of an hour or a day. Global measures of impulsivity require participants to rely on retrospective memory and gauge how they would react in “most situations”; both of these processes are riddled with cognitive biases (Shiffman et al. 2008). Moreover, retrospective reports of impulsivity do not capture the within-person variability in impulsivity that exists due to person-environment or person-situation interactions. In order to gain a fuller understanding of mechanistic relationships between, for example, impulsivity and the onset of new mood episodes, research must strive to measure impulsivity and affect with higher temporal resolution.

Several studies have explored the relationship between momentary affect and momentary impulsivity in non-bipolar-disordered clinical samples such as borderline personality disorder (Law et al. 2016; Tomko et al. 2015), alcohol users (Stamates et al. 2019), and also in non-clinical samples (Sperry et al. 2018). In general, these studies found a concurrent relationship between high negative affect and high impulsivity, although the temporal relationship between the two states remains unclear. Few studies, however, have focused on the association of momentary affect and impulsivity in BD. One pilot study compared participants with BD to healthy participants (HCs) on twice-daily EMA measures of mood and impulsivity; they found that averaged mood was significantly lower over the 14-day assessment period in participants with BD compared to HCs (Schwartz et al. 2016). They found no differences in averaged impulsivity between the two groups, although they found more variability of impulsivity in the BD group than HCs. One study (Depp et al. 2016) performed time-lagged analyses of EMA-measured impulsivity in 41 BD participants and found that EMA-measured negative affect, but not EMA-measured positive affect, predicted increases in impulsivity, which subsequently predicted decreases in positive affect. However, the EMA impulsivity measure in their study was not validated against traditional impulsivity measures. Given the role of affective instability in BD, fluctuations in impulsivity may be precursors to fluctuations in positive or negative affective states or mood episodes, or vice versa. In addition, research from neuroimaging studies suggest that affect/mood regulation and impulsivity may be linked via shared biological mechanisms, such as decreased ventrolateral prefrontal cortical activity, increased amygdala activity, and decreased functional connectivity between amygdala and prefrontal cortex (Phillips and Swartz 2014). Moreover, both impulsivity and affect appear to be regulated by dopamine transmission, and recent studies have suggested that a failure in dopamine receptor and transporter homeostasis might underlie BD and impulsivity pathophysiology (Ashok et al. 2017). Given these intriguing lines of research, it would be interesting to clarify the temporal precedence of changes in affect/mood and impulsivity, which will be critical for a better mechanistic understanding of processes in BD.

The current study sought to examine these temporal associations by measuring daily impulsivity and affect in an adult sample of individuals with BD and HCs. We sought to examine the association between different impulsivity measurements (retrospective self-report, behavioral, and daily EMA-measured), to compare impulsivity means between participants with BD and HCs, and to understand the temporal relationship between daily EMA-measured impulsivity and mood/affect in participants with BD. We hypothesized that (1) impulsivity measures would be moderately correlated with one another in the full sample, (2) individuals with BD would show higher levels of impulsivity on all measures compared to HCs, and (3) higher impulsivity would predict next-day negative affect in the BD group. Finally, we hypothesized that (4) higher variability in impulsivity over the course of the study would predict higher variability in next-day affect in the BD group.

## Methods

Data were drawn from a longitudinal study on aging, inflammation, and BD that ran from November 2016 to October 2019. 293 participants consented to the study, and 198 were eligible for assessment (113 HCs and 85 individuals with BD). Participants were excluded from participating in the study if they had acute medical illness, pregnancy, recent vaccination, history of neurological disorder, head trauma with unconsciousness > 15 min, substance abuse diagnosis in past three months or substance dependence diagnosis in past six months, history of radiation or chemotherapy treatment, or medical condition that affected inflammatory processes or ability to complete the study protocol (e.g., vision or hearing limitations, chronic pain). Because study enrollment occurred throughout the 5 year study, of the 198 eligible participants, 167 completed year one of data collection, 120 completed year two, 20 completed year three, and five completed year four. Participants in the final sample (N = 172) were 102 HCs and 70 individuals diagnosed with BD I or BD II (N = 66 and N = 4, respectively) according to the Structured Clinical Interview for the Diagnostic and Statistical Manual of Mental Disorders, fourth edition (DSM-IV) criteria (SCID; First et al. 2002). DSM-IV criteria for BD were used in an attempt to harmonize data with other studies on accelerated aging that were conducted prior to 2013 (the release date of the DSM-5). At the start of the study, 20 participants were in full remission from BD, 16 were in partial remission, 17 were currently symptomatic, 15 currently met full criteria for a (hypo)manic or depressive episode, and 2 had an unknown current mood status. Participants who had low adherence to smartphone survey completion (defined as fewer than 13 of 42 possible surveys completed in a given two-week period) were excluded from the final sample. Study procedures were approved by the University of California, San Diego Human Research Protections Program prior to start date, and all participants completed informed consent prior to beginning the study.

## Procedure

Participants completed annual assessments (bursts) over the course of 14 days that included three in-person assessments at the beginning, middle, and end of the 2-week period, and responding three times per day to self-report surveys via smartphone. EMA assessments were delivered to participants via a study-provided Samsung Galaxy 3 smartphone through a secure internet connection from a password-protected computer server controlled by MOBIT at the University of California, San Diego. Ratings were made on 14 consecutive days for a total of 42 ratings; the prompting strategy was interval-based, with surveys sent three times per day at random intervals approximating morning, afternoon, and evening. Some participants were followed over multiple years for annual 14-day data collection bursts, resulting in all participants having at least one burst of data and many having multiple (*M* number of total days of data = 23.37, *SD* = 9.13, range = 7 – 51).

## Measures

### In-person measures

#### Structured clinical interview for the DSM-IV-TR (SCID (First et al. 2002))

BD diagnoses were made at baseline using the semi-structured, clinician-administered clinical interview, designed to yield psychiatric diagnoses consistent with DSM-IV-TR criteria (American Psychiatric Association 2000) (American Psychiatric Association 2000). The interview lasts between 15 min and 2 h and contains a mixture of open-ended and scripted questions to be asked verbatim. The SCID was administered by trained clinical research staff.

#### Barratt impulsiveness scale (BIS (Patton et al. 1995))

Participants completed the BIS, a 30-item self-report measure designed to assess trait impulsiveness, once per year. The BIS is arguably the most commonly used self-report measure for assessment of impulsivity; it has strong test–retest reliability and validity (Stanford et al. 2009).

#### 5-choice continuous performance test (5C-CPT (Young et al. 2013))

The 5C-CPT (completed at the three-times-yearly in-person assessments) is a cross-species behavioral task administered via computer that assesses various aspects of attention, vigilance, and the ability to inhibit responding to non-target stimuli. The participant is given a joystick and presented with five white lines on a black screen and told that when a 2 mm white circle appears behind one of the lines, they must move the joystick in the appropriate direction to respond (target stimuli); however, if white circles appear behind every white line simultaneously, they must not respond (non-target stimuli). Thus, the task requires the participant to respond to relevant stimuli and inhibit response to irrelevant stimuli. This task has distinguished between attentional abilities in schizophrenia patients and non-psychiatric subjects in a previous study, in addition to revealing severe task deficits in BD patients with mania and moderate task deficits in BD euthymic patients (Young et al. 2020; American Psychiatric Association 2000). Although this measure has been used previously to examine differences between groups, we assessed more frequently to enhance reliability and in order to examine within-person as well as between-person changes. For measuring impulsivity or inability to inhibit a response in our study, we used total number of commission errors (i.e., number of times a participant failed to inhibit the prepotent response during the task).

#### Hamilton depression rating scale (HAM-D (Hamilton 1967))

The HAM-D, completed by BD participants at baseline, is a 17-item clinician-rated scale of depressive symptoms experienced over the past week. The measure was administered by trained clinical research staff in our study. Nine items are rated from 0 (absent) to 4 (incapacitating), and the other eight items are scored from 0 (absent) to 2 (present) given that they are difficult for the participant to classify. Higher scores indicated more severe depressive symptoms.

#### Young mania rating scale (YMRS (Young et al. 1978))

The YMRS, completed by BD participants at baseline, is an 11-item clinician-rated measure of mania symptoms experienced over the past 48 h. The measure was administered by trained clinical research staff in our study. Seven items are rated 0–4, and the remaining four (irritability, altered speech, racing thought content, and disruptive or aggressive behavior) are rated on a 0–8 Likert scale in case of poor cooperation from a subset of patients. Higher scores indicated more severe manic symptoms.

### EMA measures

#### EMA affect/impulsivity ratings

Participants completed remote ratings three times per day via mobile phone at random intervals approximating morning, afternoon, and evening. Self-reported momentary ratings (e.g., “How impulsive are you feeling right now?”) were made on 14 consecutive days during each annual burst. Ratings were made on a scale of 0–7 on the following: impulsive, sad/depressed, angry/upset, anxious/nervous, stressed, happy, energetic, and relaxed.

#### Statistical analysis plan

Given measurements occurring over time, nested within individuals, we utilized multilevel modeling, which facilitated an examination of within-person versus between-person variance. Analyses were conducted using SPSS Version 24.

In preparation for primary analyses, we tested all variables for heteroscedasticity of residuals and found that 5-choice CPT task commission errors showed significant heteroscedasticity. To correct for this, we performed a log (10) transformation and used the altered variable for all subsequent analyses.

Per factor analysis conducted by Depp and colleagues with EMA affect ratings (Depp et al. 2016), we constructed a composite measure of positive affect consisting of EMA ratings of happiness (i.e., “How happy are you feeling right now?”), energy, and relaxation. We also constructed a composite measure of negative affect using EMA ratings of sadness/depression, anxiety/nervousness, anger/upset, and stress.

For testing basic correlations between various impulsivity measures (traditional self-report, behavioral, and EMA), we used Pearson bivariate correlations. We added group (BD/HC) to the model as a second step to test whether group created a spurious correlation between impulsivity measures. Additionally, we sought to compare two associations: the association between traditional self-report impulsivity and mania/depression measures (Barratt, HAM-D, and YMRS) and the association between EMA-measured impulsivity and affect (EMA person-means of impulsiveness, positive affect, and negative affect). We used linear regressions to test these associations, controlling for demographic variables as appropriate (see below).

To test whether EMA-measured impulsivity predicted next-day affect (and vice versa), we elected to use time-lagged analyses. We lagged positive and negative affect by one day to test the effect of impulsivity (day n) on next-day positive and negative affect (day n + 1). We also tested the reverse (positive and negative affect on day n predicting impulsivity on day n + 1). Impulsivity on day n was controlled for when using positive or negative affect to predict next-day (n + 1) impulsivity, and affect on day n was controlled for when using impulsivity to predict next-day (n + 1) affect. A random intercept was included in the model to control for participant. For all time-lagged analyses, we disaggregated within-person and between-person effects per Curran and Bauer (Curran and Bauer 2011), so as not to confound within-person and between-person processes. To accomplish this, we person-mean centered the predictors (e.g., daily impulsivity and affect means) and included an aggregated person-level predictor variable in the model. The person-mean centered variable, which measured the deviation of a participant’s daily score from their own overall mean, was the measure of within-person effect. The aggregated person-level mean, which measured the deviation of one person’s overall mean from the sample mean, was the measure of between-person effect.

In addition to analyses with mean scores (described above), two measures of variability were calculated for the purposes of examining variability in impulsivity as a predictor of variability in affect and vice versa. We utilized two separate measures of variability to capture the magnitude of a person’s variation around their own mean (within-person), and to capture the magnitude of overall variability as compared to other individuals (between-person). The first within-person variability measure we calculated was atypicality, serving as a measure of how different an individual’s mean impulsivity or affect score is on a given day from that individual’s average impulsivity or affect over the entire two-week burst. We calculated atypicality by squaring the difference between a participant’s mean value and their specific day’s value (Kaufmann et al. 2016). We calculated atypicality for the EMA impulsivity measure and the EMA affect measures. A higher atypicality value represents a more abnormal impulsivity or affect rating for that person on that particular day.

The second measure of variability we calculated was the root mean square of successive difference (RMSSD). RMSSD serves as a summary score of the average amount of change that occurs from one EMA survey to the next for a specific individual; one RMSSD score was calculated per individual for each affect or impulsivity score of interest. For example, a high RMSSD impulsivity score represents a greater tendency for an individual to have large changes in impulsivity rating from one EMA survey to the next. The RMSSD score was obtained by first calculating difference scores between one survey and the next; each of those values was then squared, and the result was averaged before taking the square root of the average. We performed linear regressions to test whether the RMSSD of impulsivity predicted the RMSSD of affect, and vice versa. For all RMSSD analyses, we controlled for mean level of the corresponding predictor RMSSD variable (e.g., impulsivity mean, positive affect mean, or negative affect mean).

## Results

Participants (N = 172, M age at enrollment = 47.02, range 26–61) were 58% female, and 48.3% self-identified as white, 27.3% as Hispanic, 14.5% as African American, 4.7% as Asian, 2.3% as Native Hawaiian or other Pacific Islander, and 2.9% as multiracial or other. The average number of years of education in the sample was 15.09 (range 9–20).

Descriptive statistics of the sample are presented in Table 1.

**Table 1:** Descriptive statistics for the study participants

Participant characteristics	Total Sample, Mean (SD) or N (%)	BD group	HC group
	*N* = 172	*N* = 70	*N* = 102
Race/ethnicity (White)^†^	83 (48.3%)	41 (58.6%)	42 (41.2%)
Sex (Female)	100 (58.1%)	45 (64.3%)	55 (53.9%)
Age	47.02 (8.87)	47.40 (9.17)	46.75 (8.68)
Years of education		14.53 (2.10)	15.47 (2.11)
BD diagnosis^††^	15.09 (2.15)	–	–
*Bipolar I*	–	66 (94.2%)	–
*Bipolar II*	–	4 (5.7%)	–

^†^Race/ethnicity was self-identified

^††^BD diagnoses made according to DSM-IV-TR criteria

Means, standard deviations, and bivariate correlations of interest are presented in Table 2.

**Table 2:** Summary of variable means, standard deviations, and bivariate correlations

Measure	Mean	SD	2	3	4	5	6	7	8	9	10
1. Barratt Impulsiveness Scale Total Score	61.28	13.96	0.18*	0.29*	0.33*	0.33**	−0.39**	0.51**	0.43**	0.16	0.40**
2. 5-Choice CPT Task: Commission Errors	5.20	11.24	–	− 0.10	0.04	0.16	− 0.09	0.18*	0.14	0.18*	0.19*
3. YMRS Score (BD group only)	5.49	4.53		–	0.24*	0.31*	−0 .21	0.18	0.19	0.08	0.10
4. HAM-D score (BD group only)	18.39	10.52			–	0.22	− 0.48**	0.56**	−0 .09	−0 .11	0.19
5. EMA Impulsivity Daily Mean	1.65	0.80				–	−0.27**	0.46**	0.69**	0.29**	0.36**
6. EMA Positive Affect Daily Mean	4.77	1.07					–	− 0.67**	−0 .29**	−0 .17*	− 0.37**
7. EMA Negative Affect Daily Mean	1.86	0.89						–	0.42**	0.19*	0.59**
8. EMA Impulsivity RMSSD	0.6244	.5335							–	0.48**	0.58**
9, EMA Positive Affect RMSSD	0.765	0.324								–	0.54**
10. EMA Negative Affect RMSSD	0.586	0.371									–

*p < .05, **p < .01

Gender was associated with BIS score, such that females had a higher score than males (*t(129)* = 2.56, *p* = 0.011). Age at enrollment was positively correlated with HAM-D score, such that older age predicted higher HAM-D score (*r(65)* = 0.29, *p* = 0.017). Race/ethnicity was associated with 5C-CPT total commission errors (African American participants had higher number of errors on average than white participants, *F(6, 163)* = 3.44, *p* = 0.003), daily mean of positive affect composite (African American participants had significantly lower daily average positive affect than white participants, *F(6, 148)* = 2.56, *p* = 0.022), and RMSSD of positive affect (biracial participants had significantly higher RMSSD than white, African American, or Hispanic/Latinx participants, *F(6, 147)* = 2.57, *p* = 0.022). It is possible that associations between self-identified race/ethnicity and 5C-CPT/positive affect score are due to various social determinants (e.g., socioeconomic status, discrimination) which were not measured in this study. Thus, drawing conclusions from these results should be done with caution. Due to these observed associations, we controlled for gender in all analyses where BIS score was the dependent variable, for age at enrollment when HAM-D was the dependent variable, and for race/ethnicity when 5C-CPT commission errors, daily mean of positive affect, or RMSSD of positive affect were the dependent variables.

### Correlations between impulsivity measures

Pearson bivariate correlation coefficients were computed to assess the linear relationships between different measures of impulsivity: traditional self-reported impulsivity (BIS score) EMA-measured impulsivity (“How impulsive are you feeling right now?”), and behavioral impulsivity (5C-CPT total commission errors). There was a positive correlation between EMA-measured impulsivity rating and BIS total score, *r*(124) = 0.33, *p* < 0.001, but not between EMA-measured impulsivity rating and 5C-CPT commission errors. BIS score was also positively correlated to 5C-CPT total commission errors, *r*(131) = 0.18, *p* = 0.036. When group (BD and HC) was added to the model as a predictor, the relationship between BIS score and EMA impulsivity was still significant (*F*(3,120) = 34.20, *p* < 0.001, *t* = 2.45, *p* = 0.016), but the relationship between BIS score and 5C-CPT task was no longer significant (*F*(3,127) = 37.17, *p* < 0.001, *t* = 1.00, *p* = 0.318).

### Group differences in impulsivity

To compare impulsivity in the BD versus HC groups, four linear mixed models were conducted. Participants in the BD group had significantly higher BIS total score (*t(122.92)* =  − 9.72, *p* < 0.001), higher EMA impulsivity daily score (*t(155.69)* = − 4.23, *p* < 0.001), higher EMA daily atypicality (*t(152.04)* =  − 5.06, *p* < 0.001), and higher number of commission errors (*t(151.64)* =  − 3.31, *p* = 0.001) than those in the HC group.

### Person-level associations between impulsivity and affect

Linear regressions were also conducted in the BD group to measure the association between traditional measures of mood and impulsivity (BIS score, YMRS, and HAM-D), and to measure the corresponding association between EMA-measured affect and impulsivity. High HAM-D-measured depression was associated with high BIS-measured impulsivity, *F*(2,52) = 7.25, *p* < 0.001, *t* = 3.08, *p* = 0.004 (Cohen’s *f*^*2*^ = 0.16), and correspondingly, high EMA negative affect score was associated with high EMA impulsivity score, *F*(1,60) = 10.75, *t* = 3.28, *p* = 0.002 (Cohen’s *f*^*2*^ = 0.18). For mania/positive affect, high YMRS-rated mania was associated with high BIS-measured impulsivity, *F*(2,52) = 6.61, *p* = 0.003, *t* = 2.84, *p* = 0.006 (Cohen’s *f*^*2*^ = 0.14). However, there was no significant correlation between the EMA positive affect and the EMA impulsivity item score, *F*(1,60) = 1.25*, t* = − 1.12, *p* = 0.269.

### Time-lagged models of daily impulsivity and affect

Results of time-lagged LMMs indicated that daily positive affect was not significantly associated with daily impulsivity. However, higher daily negative affect significantly predicted higher daily impulsivity over the course of the study (*t(50.92)* = 3.496, *p* < 0.001). Conversely, higher mean impulsivity significantly predicted higher negative affect over the course of the study (*t(49.26)* = 3.423, *p* < 0.001).

We ran a series of exploratory post-hoc analyses to examine which components of positive affect (i.e., happiness, energy, and relaxation) and negative affect (i.e., sadness, anger/upset, nervousness/anxiety, and stress) were driving the significant association with impulsivity. Only the happiness component of positive affect seemed to predict lower impulsivity (*t* (51.54) = − 2.029, *p* = 0.048) and vice versa (*t* (48.39) = − 2.037, *p* = 0.029), whereas energy and relaxation were not significantly associated with impulsivity. All components of negative affect were significantly associated with higher next-day impulsivity, but the angry/upset item and the anxious/nervous item had the strongest relationship to impulsivity (*t (50.78)* = 4.873, *p* < 0.001 and *t(52.03)* = 3.820, *p* < 0.001, respectively). Similarly, when we reversed the direction of predictors, impulsivity seemed to predict all components of negative affect, but angry/upset and nervous/anxious affect states were most strongly predicted (*t (53.34)* = 4.992, *p* < 0.001 and *t (52.37)* = 3.665, *p* < 0.001). Notably, some within-person changes were significant when impulsivity was used to predict next-day affect: an individual’s level of impulsivity significantly predicted higher next-day anger/upset (*t (1386.89)* = 2.273, *p* = 0.023) and higher next-day anxiousness/nervousness (*t (1839.53)* = 2.829, *p* = 0.005), but the converse was not true. Thus, increases in impulsivity appear to temporally precede increases in anger/anxiety even while accounting for same-day anger/anxiety.

### Variability of impulsivity and affect

Atypicality (variability) of both negative and positive affect significantly predicted higher atypicality of impulsivity (*t(55.69)* = 5.95,* p* =  < 0.001 and *t(65.79)* = 5.22, *p* < 0.001, respectively), and conversely, higher atypicality of impulsivity predicted higher atypicality of negative and positive affect (*t(65.87)* = 5.50, *p* < 0.001 and *t (59.70)* = 5.33, *p* < 0.001, respectively).

As a parallel analysis to the atypicality analyses, we tested whether RMSSD of positive affect predicted an individual’s RMSSD of impulsivity, controlling for mean levels of positive affect, and found a significant effect (*F*(2,59) = 10.75, *p* < 0.001, *t* = 4.548, *p* < 0.001, Cohen’s *f*^*2*^ = *0.3*5). Similarly, we found that RMSSD of negative affect significantly predicted RMSSD of impulsivity (*F*(2,59) = 16.22, *p* < 0.001, *t* = 5.253, *p* < 0.001, Cohen’s *f*^*2*^ = *0.4*3). Thus, these analyses seemed to follow the same pattern as the atypicality analyses summarized above.

## Discussion

In summary, we validated a one-item EMA measure of impulsivity, confirming its association with a traditional measure and demonstrating that it accurately distinguishes BD and HC participants. Moreover, we found a bidirectional relationship between daily levels of negative affect and impulsivity; parsing this relationship revealed that increased impulsivity led to increased next-day anger and anxiety. We also found that increases in affective variability were bi-directionally related to increases in variability of impulsivity.

Our one-item EMA measure of impulsivity was validated against a traditional self-report measure of impulsivity and a behavioral measure of impulsivity; the EMA impulsivity measure was significantly correlated to the BIS (*r* = 0.33) but not 5C-CPT commission errors (*r* = 0.18). This study supports our EMA impulsivity measure as a valid alternative for a traditional retrospective self-report measure, and supports the premise that momentary measures add value and nuance to our research by virtue of capturing within-person changes over time. Our findings align with previous studies that have found EMA impulsivity measures correlate moderately with their retrospective facsimiles (Sharma et al. 2013; Halvorson et al. 2021; Tomko et al. 2015).

Compared to HCs, the BD group had higher self-reported impulsivity, higher behavioral impulsivity, higher EMA-measured impulsivity, and higher variability (atypicality) of EMA-measured impulsivity. Our work is consistent with past literature establishing that impulsivity is generally elevated in BD as compared to HCs (Strakowski et al. 2010; Swann et al. 2009). Our findings demonstrate the predictive validity of the EMA impulsivity measurement and add further support to the predictive validity of the BIS and 5C-CPT commission errors.

Furthermore, our time-lagged analyses revealed a bidirectional association between mean levels of negative affect and impulsivity (but not positive affect) over the course of the study. Finally, with respect to the variability of affect, we found that greater variability (atypicality) of both positive and negative affect predicted greater next-day variability of impulsivity; this was also true in the reverse, such that impulsivity variability predicted next-day positive and negative affect variability. It follows that next-day affect may be less predictable in participants who have highly unstable impulsivity levels. Given that our analyses allowed for separation of between-person from within-person effects, a clear result of our study was that between-person effects seemed to be driving the associations between affect and impulsivity far more strongly than within-person effects. Essentially, a participant who was generally high in impulsivity tended to be generally high in negative affect and low in positive affect over the course of the study, rather than day-to-day changes in impulsivity driving next-day changes in affect. However, post hoc analyses revealed an exception: impulsivity was a significant within-person driver of next-day anger, upset, anxiousness, and nervousness.

The reported findings align with studies of non-bipolar-disordered samples that found a strong association between daily impulsivity and negative affect (Sperry et al. 2018; Law et al. 2016; Tomko et al. 2015). However, with respect to the temporal interplay between affect and impulsivity, our findings differ slightly from a previous study (Depp et al. 2016). Whereas Depp and colleagues (Depp et al. 2016) found that negative affect predicted impulsivity, which subsequently predicted lowered positive affect, we found a bidirectional relationship between impulsivity and negative affect, and no significant relationship between positive affect and impulsivity. Moreover, our post hoc analyses found that impulsivity predicts certain types of next-day negative affect (e.g., anger, anxiety) rather than negative affect preceding impulsivity. Given these mixed findings, the day-to-day causal relationship between affect and impulsivity in BD remains unclear. The current study distinguished between within-subject and between-subject lagged effects, whereas the 2016 study did not, which may account for some of the discrepancies in findings. Future studies should use nuanced measurement and analysis methods to further explore these complex relationships.

Our study evinced several notable strengths, including a large, well-characterized sample of individuals with BD, a healthy comparison group, and detailed information about the participants’ mood state at the time of assessment. We used several measures of impulsivity employing varied methodologies (i.e., self-report, behavioral, and EMA), which allowed us to characterize this multi-faceted construct with considerable nuance. Moreover, our intensive longitudinal measurement of impulsivity created a rich dataset that permitted powerful time-lagged analyses of within-person changes, enabling our exploration of the temporal interplay between affect and impulsivity. This is only the second study, to our knowledge, to examine the interlinkage of impulsivity and affect in BD using an EMA paradigm, and the first study to also examine the validity of the mobile impulsivity measure.

Despite these strengths, the current study is limited by several factors. Our EMA impulsivity measure only consisted of one item (“How impulsive are you feeling right now?”), and although it has high face validity, it may not capture the impulsivity construct in its totality. Thus, this study represents only a preliminary examination of EMA-based impulsivity and affect. Future research might address this issue by thoughtfully constructing a multi-item measure of momentary impulsivity and thoroughly validating it against existing measures. Although EMA is an exciting methodology with numerous benefits, a few inherent drawbacks exist, such as the potential for participant reactivity to receiving multiple surveys per day and the variable level of participant comfort with using smartphone technology. A final limitation: it may be that impulsivity’s strong relation to negative affect is due to their belonging to a shared higher-order factor (e.g., neuroticism, general distress level) which was not measured or controlled for in the current study. Future research might seek to understand the temporal dynamics of impulsivity and negative affect above and beyond person-level variables (e.g., neuroticism) by controlling for these personality factors in the analysis.

## Conclusions

BD researchers have long wondered whether fluctuations in day-to-day impulsivity can lead to changes in mood or affect–an important potential predictor given previous findings that shifts in affect can precipitate onset of a manic/depressive episode in BD (Meter et al. 2016). The results of the present study indicate that increased impulsivity predicts increased next-day anger, upset, anxiousness, and nervousness in a sample of participants with BD. An ecological momentary assessment perspective on the interplay between impulsivity and affect in BD will allow us to better understand the mechanisms driving mood symptom and episode onset, which could ultimately increase predictability of mood episodes and suggest new person-specific prevention methods or treatments. For instance, for a specific individual, elevated levels of impulsivity compared to their average may indicate a high-risk period for developing negative affect or depressive symptoms, and creating detection systems for monitoring and suggesting preemptive measures before mood decreases could prevent the onset of new depressive episodes.

## Data Availability

Portions of the data that support the findings of this study are openly available in the NIMH data archive at: https://nda.nih.gov/edit_collection.html?QA=false&id=2719, reference number 2719. The remaining data that support the findings of this study are available from the corresponding author upon reasonable request.

## Abbreviations

5C-CPT: 5-Choice continuous performance task
BD: Bipolar disorder
BIS: Barratt Impulsiveness Scale
DSM-IV: Diagnostic and Statistical Manual of Mental Disorders, fourth edition
DSM-5: Diagnostic and Statistical Manual of Mental Disorders, fifth edition
EMA: Ecological momentary assessment
HAM-D: Hamilton Depression Rating Scale
HC: Healthy comparisons
LMM: Linear mixed model
RMSSD: Root mean square of successive difference
SCID: Structured Clinical Interview for the DSM-IV
YMRS: Young Mania Rating Scale
